# Perioperative Enteral Immunonutrition Support for the Immune Function and Intestinal Mucosal Barrier in Gastric Cancer Patients Undergoing Gastrectomy: A Prospective Randomized Controlled Study

**DOI:** 10.3390/nu15214566

**Published:** 2023-10-27

**Authors:** Mingwei Ma, Zicheng Zheng, Ziyang Zeng, Jie Li, Xin Ye, Weiming Kang

**Affiliations:** Department of General Surgery, Peking Union Medical College Hospital, Chinese Academy of Medical Science and Peking Union Medical College, Beijing 100730, China; 13324593358@163.com (M.M.); zh.z.c@163.com (Z.Z.); pumc_ziyangzeng@student.pumc.edu.cn (Z.Z.); pumc_jieli@student.pumc.edu.cn (J.L.); yexinpumch@163.com (X.Y.)

**Keywords:** immunonutrition, perioperative period of gastric cancer, intestinal mucosal barrier, immune function, inflammatory response

## Abstract

Objective: The impact of perioperative immunonutrition on patients undergoing radical gastrectomy remains undetermined. This study aimed to assess the influence of enteral immunonutrition support on postoperative immune function and intestinal mucosal barrier function following radical gastrectomy, contrasting findings with a control group to furnish evidence for perioperative enteral nutrition support. Methods: In this prospective randomized trial, 65 patients who underwent radical gastrectomy between June 2022 and June 2023 were included. Participants were allocated to either the study group (receiving enteral immunonutrition) or the control group (not receiving enteral immunonutrition). We compared postoperative rehabilitation and complications between the groups, analyzed the intestinal mucosal barrier function markers on the 3rd and 7th postoperative days, and delved deeper into peripheral blood cell immunity, inflammation, and nutritional indicators. Results: The cohort consisted of 30 patients in the study group and 35 in the control group, with no significant differences in demographic attributes between the two groups. On the 3rd postoperative day, the diamine oxidase, D-lactic acid, and endotoxin levels in the study group were significantly lower than those in the control group (*p* = 0.029, *p* = 0.044, and *p* = 0.010, respectively). By the 7th postoperative day, these levels continued to be significantly diminished in the study group (*p* = 0.013, *p* = 0.033, and *p* = 0.004, respectively). The times to first flatus (*p* = 0.012) and first bowel movement (*p* = 0.012) were significantly shorter in the study group. Moreover, postoperative complications in the study group were fewer than in the control group (*p* = 0.039). On the 7th postoperative day, the study group had lower peripheral white blood cell (WBC) levels (*p* = 0.020) and neutrophil–lymphocyte ratios (NLR) (*p* = 0.031), but displayed elevated albumin levels (*p* = 0.006). One month post-surgery, the CD4+T and CD8+T counts were significantly greater in the study group (*p* = 0.003 and *p* = 0.012, respectively). Correlation analyses indicated that NLR and complications were associated with endotoxin levels. Conclusion: Administering perioperative enteral immunonutrition enhances postoperative immune and intestinal mucosal barrier functions in patients undergoing radical gastrectomy. This effect leads to diminished inflammatory responses, a decreased rate of postoperative complications, and accelerated patient recovery.

## 1. Introduction

Gastric cancer stands as one of the predominant malignancies of the gastrointestinal system. According to the Global Cancer Statistics 2020, it is the fifth most common cancer globally regarding incidence, and is ranked fourth in mortality [1]. With a H. pylori prevalence rate of 44.2% in China, gastric cancer manifests with a notably high incidence, presents therapeutic challenges, and often culminates with an unfavorable prognosis, gravely imperiling the health of individuals [2,3]. Currently, surgical resection remains the first-line treatment for gastric cancer [4]. Literature underscores that patients diagnosed with gastric cancer often exhibit marked attenuation in both cellular and humoral immunity, concurrently with heightened malnutrition risk [5,6]. The intestinal mucosal barrier, instrumental in precluding deleterious enteric substances from accessing systemic circulation, is a conglomerate of chemical, mechanical, biological, and immunological defenses closely related to the systemic inflammatory response [7,8]. The physiological stress instigated by radical gastrectomy can subdue systemic immune responses and concurrently induce intestinal mucosal ischemia and hypoxia, coupled with alterations in the intestinal microbial composition. Such disturbances may escalate postoperative complications and impinge upon long-term patient survival [9]. Emerging evidence postulates that the incorporation of immunonutrients during the perioperative period of patients with gastrointestinal cancer can bolster the nutritional status, protect the intestinal mucosal barrier, mitigate inflammatory surges, recalibrate immunity, and consequently exhibit anti-tumor effects while curbing complications [10,11,12]. Previous studies have not analyzed intestinal mucosal barrier indexes to show the advantages of enteral immunonutrition.

In this pioneering endeavor, we gauge the ramifications of perioperative enteral immunonutrition (EIN) administration on postoperative recovery trajectories following gastric cancer surgery by meticulously assessing indicators reflective of the function of the intestinal mucosal barrier. We juxtapose these findings against a control group receiving enteral nutrition (EN) devoid of immunonutrients. Our overarching objective is to discern the differential impacts of these nutritional support treatments on postoperative inflammatory response, immune function, and intestinal barrier function, thereby crafting an evidence-based foundation for perioperative nutritional interventions in gastric cancer.

## 2. Materials and Methods

### 2.1. Study Participants

This study is a single-center, prospective, randomized investigation aimed at assessing the efficacy of perioperative immunonutrition in patients undergoing radical gastrectomy for gastric cancer. The cohort encompassed patients who underwent radical gastrectomy from June 2022 to June 2023 at the Peking Union Medical College Hospital.

Inclusion Criteria: 1. Pathologically confirmed diagnosis of gastric cancer; 2. Complete clinical data; 3. Aged 18–80 years; 4. Absence of radiotherapy or chemotherapy interventions within the four weeks before surgery; 5. Patient’s informed consent and signature on the consent form.

Exclusion Criteria: 1. Manifestation of severe clinical symptoms or comorbidities; 2. Diagnoses of other concurrent malignancies; 3. Patients who are pregnant or lactating; 4. Inability to take oral or enteral nutrition during the perioperative period; 5. Perioperative administration of fat emulsions enriched in *n*-3 fatty acids.

**Figure 1:**
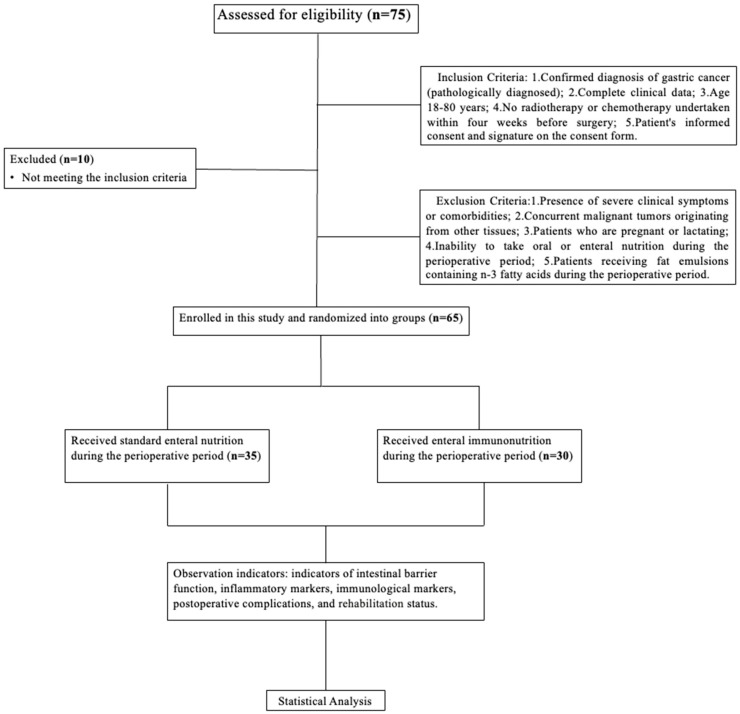
The flowchart of our study.

Based on the chosen perioperative nutritional support, patients were randomized into either the study group (benefiting from enteral nutrition supplemented with immunonutrients) or the control group. This study strictly adhered to the ethical principles of medical research involving patients, as laid out in the Declaration of Helsinki. Ethical clearance for the study was approved by the ethics committee of the Peking Union Medical College Hospital, documented under the code KS2022530 and dated 9 February 2022. All enrolled participants were included in the research after a detailed explanation of the study protocol and their written informed consent, or that of their family members was procured. The flowchart of our study is shown in Figure 1.

### 2.2. Methods of Perioperative Nutritional Support for Both Groups

This investigation was a prospective, randomized clinical trial conducted at the Peking Union Medical College Hospital. Patients were randomized to one of two enteral nutrition protocols. Entenal immnuonutrition (PROSURE group: EIN, n = 30) was administered in the first group, a liquid diet providing 1.26 kcal/mL and 6.65 g of protein per 100 mL. This diet is supplemented with *n*-3 fatty acids (4.6 g/L) and dietary fiber (2.07 g/100 mL). The second group received enternal nutrition (Ensure group: EN, n = 35), a standard enteral feed delivering 1.5 kcal/mL and 6.25 g of protein per 100 mL, devoid of specialized immunonutrients. The patients received enteral nutrition associated with their regular meals from the 4th day preceding surgery to the day before surgery, and enteral nutrition from the 3rd to the 14th day postoperatively. The daily caloric provision from enteral nutrition was established at 20 kcal/kg/day. The volume of the enteral formula consumed, alongside oral food intake, was meticulously monitored preoperatively by a dedicated researcher for all participants. A blinded envelope approach was employed for randomization: an equivalent number of envelopes, each containing either the EIN or IN protocol, were prepared without indication of their content. These envelopes were then sequentially dispensed as patients were inducted into the study. This random allocation process was executed five days before surgery, ensuring that intraoperative observations had no bearing on the group assignment.

All participants were counseled to ingest an oral enteral supplement, equivalent to 20 kcal/kg/day, in tandem with their regular diet, commencing on the fourth day before surgery and continuing until surgery eve. Postoperative enteral nutrition was also administered from the 3rd to the 14th day after surgery. Enteral nutrition was started and increased progressively each day in a stepwise manner. This postoperative enteral nutrition was sustained even after the resumption of oral intake until approximately 14 days after surgery. The blood specimens were drawn from a peripheral vein approximately five days before surgery (baseline), then at postoperative intervals of 3 and 7 days, and again at one month.

### 2.3. Detection of Observation Indicators

The primary endpoint was postoperative complications. The secondary endpoints encompassed parameters such as nutritional status, inflammatory markers, cellular immune function indicators, and intestinal mucosal barrier integrity indicators, notably endotoxin (ET), D-lactic acid, and diamine oxidase. Peripheral venous blood samples, procured after an 8 h fasting period pre-surgery and on the 3rd and 7th postoperative days were assessed for the abovementioned intestinal mucosal barrier indicators. Inflammatory markers, including total white blood cell counts, the neutrophil–lymphocyte ratio (NLR), and nutritional markers like albumin were evaluated on the 7th postoperative day. The indicators of cellular immune function, CD4+T and CD8+T, were assessed preoperatively and one month postoperatively. The incidence of postoperative complications and gastrointestinal metrics, such as the time to the first postoperative flatus and bowel movement in both groups, were also documented.

### 2.4. Evaluation Indicators

Comparisons were undertaken between the groups concerning demographic data, postoperative rehabilitation, postoperative complications (Clavien-Dindo ≥ 2), and indicators of the intestinal mucosal barrier on the 3rd and 7th postoperative days, including D-lactic acid, diamine oxidase, and endotoxin. Peripheral blood inflammatory markers, including white blood cell (WBC) counts and NLR, and nutritional indicators like albumin on the 7th postoperative day were contrasted between the two groups. The cellular immunity indicators, CD4+T, CD8+T, and CD4+/CD8+ ratio one month postoperatively, were compared.

### 2.5. Statistical Methods

The primary endpoint of the study was complications after gastrectomy. The anticipated non-occurrence rate for complications was projected at 80%, and its compared group was 60%. With an 80% statistical power and a one-sided type I error rate of 5%, a sample size of 21 patients was determined using Simon’s two-stage design. Factoring in an anticipated 10% ineligibility rate, the estimated total sample size was set at 23 patients.

SPSS software v23.0 was used for statistical analysis in this study. Categorical data were subjected to the chi-square test, while continuous data, expressed as the mean ± standard deviation (SD), were assessed using independent-sample *t*-tests; a *p*-value < 0.05 denoted statistical significance. Pearson’s product–moment correlation coefficient was used to measure the degree of correlation, with a coefficient closer to 1 indicating a stronger correlation. Pearson coefficients < 0.3 indicated weak correlations, 0.3 and 0.6 implied moderate, and >0.6 signified strong correlations. Correlation analysis was performed between complications, NLR levels, and endotoxin levels.

## 3. Results

### 3.1. General Information

Sixty-five patients undergoing radical gastrectomy for gastric cancer were included in this study, with 30 in the study group and 35 in the control group. Table 1 illustrates comprehensive patient data. No significant disparities were identified between the groups concerning gender, age, BMI, tumor staging, or surgical methods (*p* > 0.05). The preoperative indicators of cellular immune function (CD4+T and CD8+T levels) were not significantly different between the groups (*p* > 0.05). Both groups demonstrated consistent preoperative intestinal mucosal barrier function, experienced no severe postoperative complications, and no perioperative mortalities; all patients were subsequently discharged.

### 3.2. Comparison of Postoperative Indicators of the Intestinal Mucosal Barrier Function between the Two Groups

Table 2 presents comparative data on diamine oxidase, D-lactic acid, and endotoxin levels preoperatively and on the 3rd and 7th postoperative days. Preoperative measurements revealed no significant differences between the groups for diamine oxidase (t = −1.572, *p* > 0.05), D-lactic acid (t = −1.950, *p* > 0.05), and endotoxin levels (t = −1.709, *p* > 0.05). However, the study group exhibited notably reduced diamine oxidase levels on both the 3rd (t = −2.244, *p* < 0.05) and 7th (t = −2.583, *p* < 0.05) postoperative days relative to the control group. Similarly, the D-lactic acid levels in the study group were markedly lower on the 3rd (t = −2.051, *p* < 0.05) and 7th (t = −2.192, *p* < 0.05) postoperative days. Endotoxin levels followed suit, being significantly lower in the study group on the 3rd (t = −2.645, *p* < 0.05) and 7th (t = −3.027, *p* < 0.05) postoperative days.

### 3.3. Comparison of Postoperative Rehabilitation between the Two Groups

The intervals to the first flatus and first bowel movement for both groups are detailed in Table 3. Regarding postoperative recovery, the time to first flatus and first bowel movement averaged 2.88 ± 0.83 days and 4.40 ± 1.15 days, respectively, in the study group. In contrast, these measures were 3.57 ± 1.24 days and 5.51 ± 1.90 days in the control group. Notably, patients in the study group experienced significantly expedited times to both the first flatus (t = −2.578, *p* < 0.05) and first bowel movement (t = −2.605, *p* < 0.05) compared to the control group.

### 3.4. Comparison of Postoperative Complications between the Two Groups

Table 4 delineates complications (Clavien-Dindo ≥ 2) assessed across the groups using the chi-squared test. The study group manifested a markedly reduced complication rate relative to the control group (chi-square = 4.252, *p* < 0.05). Postoperative complications for the study group encompassed three instances of anastomotic leakage and two occurrences of bleeding. In contrast, the control group manifested complications such as six instances of bleeding, four anastomotic or residual fistula, three postoperative infections, and a single case of pulmonary embolism. Additionally, the correlation between complications and endotoxin levels was probed, with the correlation coefficients for diamine oxidase and endotoxin on the 3rd postoperative day, with complications being 0.335 and 0.445, respectively, and statistically significant at *p* < 0.01 (Table 4).

### 3.5. Comparison of Postoperative Inflammatory Indicators between the Two Groups

Table 5 portrays the comparison of the neutrophil-to-lymphocyte ratio (NLR) and peripheral WBC between the two groups on the 7th postoperative day. The study group manifested significantly diminished WBC counts and NLR compared to the control group (t = −2.390, *p* < 0.05; t = −2.210, *p* < 0.05, respectively). A correlation analysis of the endotoxin levels and NLR on the 7th postoperative day produced a correlation coefficient of 0.248, significant at the 0.05 threshold, indicating a significant low-grade positive correlation between postoperative endotoxin levels and NLR.

### 3.6. Comparison of Postoperative Nutritional Indicators between the Two Groups

On the 7th postoperative day, as depicted in Table 6, the albumin level in the study group was significantly elevated in contrast to the control group (t = 2.874, *p* < 0.05).

### 3.7. Comparison of Postoperative Indicators of Cellular Immune Function between the Two Groups

Table 7 presents a comparative analysis of CD4+T and CD8+T levels one month post-surgery. The study group displayed notably elevated levels of both CD4+T (t = 3.050, *p* < 0.05) and CD8+T (t = 2.594, *p* < 0.05) compared to the control group. Nonetheless, the CD4+T/CD8+T ratio demonstrated no significant variation between the groups.

## 4. Discussion

Gastric cancer, a predominant digestive system malignancy, frequently emerges with late diagnoses and challenging therapeutic measures in China. Modern therapeutic paradigms underscore a comprehensive treatment anchored in surgical interventions [13]. Alarmingly, upward of 80.4% of gastric cancer patients are at a risk of malnutrition, and surgical interventions can further deteriorate nutritional statuses, which are closely intertwined with patient prognoses [5]. Coupled with malnutrition, patients often grapple with dampened immune responses, systemic inflammatory stress responses, and compromised cellular and humoral immunity during the perioperative period. Therefore, perioperative nutritional reinforcement becomes paramount. Evidence points toward immunonutrition as a potent avenue for modulating metabolic and immune trajectories [14]. The European Society for Clinical Nutrition and Metabolism (ESPEN) posits that patients undergoing upper gastrointestinal cancer surgeries should opt for enteral immunonutrition to curtail significant infectious complications [15]. Numerous inquiries indicate that EIN therapy can attenuate postoperative complications and inflammatory manifestations, curtailing hospital durations and bolstering the patient quality of life [12,16,17]. However, debates persist concerning the relative superiority of EIN over EN in clinical and immune function indicators [16,18]. Our study delved into postoperative complications, intestinal mucosal barrier function indicators, and immune parameters across our cohorts, shedding light on the intricate interplay between EIN, intestinal barriers, and inflammatory responses, thereby offering insights for perioperative nutritional guidance.

The intestinal mucosal barrier serves a dual role: it facilitates the transport of nutritional substances from food and simultaneously obstructs detrimental substances within the intestinal cavity. This multifaceted barrier encompasses mechanical, chemical, biological, and immune barriers. The integrity of the intestinal barrier is intrinsically linked to various pathologies, including cancer [7]. Furthermore, mounting evidence underscores the association of the intestinal mucosal barrier with diverse inflammatory conditions. Surgical interventions or tumor-induced damage to this barrier, resulting in heightened permeability, can precipitate a systemic inflammatory response characterized by the activation of numerous inflammatory cells and the release of cytokines such as IL-1 and TNF-α [19,20,21]. Literature underscores that gastrointestinal surgeries can amplify intestinal permeability, with the extent of mucosal barrier damage being strongly associated with postoperative complications [22]. Hence, preserving the functionality of the intestinal mucosal barrier can mitigate systemic and intestinal postoperative complications and dampen the body’s inflammatory response. Currently, D-lactic acid is often gauged to assess intestinal permeability [10], while intestinal ischemia and mucosal damage lead to increased diamine oxidase (DAO) levels [23]. Furthermore, disruptions in the biological barrier, leading to bacterial translocation, can markedly surge serum endotoxin levels [24].

DAO, a potent intracellular enzyme abundant within intestinal villi, serves as a barometer for both the integrity of the intestinal mucosa and the extent of its damage. Moreover, a significant correlation exists between plasma DAO and intestinal mucosal DAO, rendering it a reliable marker for intestinal mucosal injury [23]. Our investigation revealed that perioperative Enteral Immune Nutrition (EIN) administration notably reduced DAO levels on the 3rd and 7th postoperative days compared to the control group. This result suggests that immunonutrients might mitigate damage to the intestinal mucosal barrier, thereby fortifying intestinal epithelial cells. It is well-documented that intestinal ischemia can lead to endotoxemia, igniting an inflammatory response and bolstering TNF-α synthesis [24,25]. In our study, endotoxin levels in the EIN group markedly decreased on the 3rd and 7th postoperative days compared to the control group. This result intimates that immunonutrition aids in attenuating intestinal mucosal damage, conserving the integrity of the epithelial structure, and curtailing endotoxin levels. Furthermore, the observed correlation between endotoxin levels and the NLR inflammatory marker indicates that systemic inflammation can be reduced by bolstering the intestinal mucosal barrier. D-lactic acid, a metabolite produced by the gut microbiota, escalates in circulation when the intestinal barrier is compromised due to malignancies, surgical interventions, or infections, causing increased permeability. Furthermore, a direct correlation exists between plasma D-lactic acid and endotoxin levels [26]. Our findings showcased diminished postoperative D-lactic acid concentrations in the study group compared to the control group, suggesting that enteral immunonutrition can augment intestinal mucosal permeability and curtail D-lactic acid entry into the bloodstream. Previous literature has affirmed that immunonutrition can refine intestinal mucosal permeability, diminish ischemia and mucosal damage, and substantially reduce postoperative complications [27].

In line with this, it has been previously elucidated that the degree of intestinal mucosal permeability and barrier damage was closely correlated to postoperative complications [27]. In the present study, we observed a noteworthy moderate, positive correlation between postoperative complications, diamine oxidase levels on the 3rd postoperative day, and intracellular endotoxin levels. These findings indicate a pronounced association between postoperative complications and disruptions in the intestinal mucosal barrier. They further suggest that enteral immunonutrition could potentially mitigate postoperative complications by enhancing the function of the intestinal mucosal barrier. Our data demonstrated a hastened time to the first flatus and bowel movement in the study group after surgery, suggesting an expedited recovery of intestinal function in these patients. Such outcomes underscore the potential of immunonutrients in minimizing postoperative complications and catalyzing postoperative rehabilitation by bolstering the intestinal barrier function. Serving as a nuanced marker of the inflammatory response [28], the NLR revealed that individuals administered with immunonutrients exhibited attenuated levels of peripheral WBC and NLR compared to the control group, suggesting a diminished inflammatory milieu in these patients. These observations further confirm that immunonutrients can temper systemic and intestinal inflammation levels, reducing predisposition to systemic inflammatory response syndrome. Regarding nutritional indicators, the albumin (ALB) level in the study group surpassed that observed in the control group, suggesting that immunonutrients might be instrumental in ameliorating malnutrition.

Historical literature indicates that diverse stressors, such as tumors, surgical interventions, chemotherapy, and physiological stress, can compromise the intestinal barrier, culminating in immune dysregulation [29]. T cells, pivotal effector cells in immunological cascades, orchestrate cellular immune responses and are indispensable for holistic immune defense mechanisms. Both CD4+ and CD8+ T cells are integral constituents of cellular immunity. CD4+/CD8+ refers to the ratio of the number of CD4+ T cells and CD8+ T cells in peripheral blood. As an index of immune regulation, the ratio of CD4+/CD8+ indicates the dysfunction of cellular immunity. In this study, the number of CD4+ and CD8+ T cells in the study group was significantly elevated in the control group, suggesting that enteral nutrition fortified with immunonutrients can recalibrate immune imbalances, foster the proliferation of CD4+ T cells, and expedite the restitution of comprehensive immune function. Previous research posits that the abundance of peripheral CD4+ T cells may prognosticate outcomes in patients afflicted with gastrointestinal tumors [30] and is closely associated with the efficacy of immunotherapy [31]. While a depressed CD4+/CD8+ T cell ratio is emblematic of immune suppression, some literature suggests that an elevated CD4+/CD8+ T cell ratio in peripheral circulation might presage favorable 5-year overall survival rates [32]. However, our study did not discern significant disparities in the CD4+/CD8+ ratio between the study (enteral immunonutrition) group and the control (enteral nutrition) group, which might be attributable to our study’s relatively constrained sample size.

The principal constituents of the immunonutrition administered in our investigation comprised fish oil-derived polyunsaturated fatty acids (PUFAs) and dietary fiber. Extant literature demonstrates that N-3 PUFAs exhibit anti-inflammatory, immunopotentiating, and immune-modulatory effects, chiefly suppressing inflammatory cytokine production [33]. Specific N-3 PUFAs, such as docosahexaenoic acid (DHA), influence neurotransmitter secretion, while eicosapentaenoic acid (EPA) competes with arachidonic acid for enzymes like cyclooxygenase and lipoxygenase. This competition leads to the genesis of anti-inflammatory molecules like prostaglandin E3 (PG-3) and leukotriene B5 (LT-5), consequently attenuating platelet-activating factor (PAF) release and the synthesis of inflammatory markers such as TNF-α and IL-1β, thereby diminishing endotoxin levels [34]. Several investigations underline EPA’s capacity to curtail IL-6 secretion, induced by endotoxins and IL-1, to stabilize cellular membranes and orchestrate various immune pathways, endorsing its anti-inflammatory and immune-regulating effects [35]. A study advocating for perioperative N-3 PUFA administration in gastric cancer patients revealed a marked reduction in the incidence of systemic inflammatory response syndrome alongside a concomitant decrease in hospitalization duration [36]. A meta-analysis postulated that N-3 PUFAs could attenuate postoperative complications and short-term mortality, rejuvenate immune functionalities in gastric cancer patients, curtail inflammatory reactions, and foster prompt recovery [37]. As a prebiotic, dietary fiber, conceptualized as an ecological immunonutrient, fortifies the intestinal biological barrier. This effect is achieved by fostering the proliferation of beneficial bacterial populations, ensuring a harmonious intestinal microbiome, facilitating intestinal epithelial cell repair, and minimizing bacterial translocation. The concomitant employment of prebiotics and N-3 PUFAs in immunonutritional regimens augments the structural and functional integrity of the intestinal mucosal barrier [38]. Research suggests that the early introduction of EIN after surgery curtails CRP and TNF-αsynthesis, markedly enhancing immune responsiveness and suppressing inflammation in patients with gastric cancer [36]. A compendium of clinical trials affirms EIN’s capacity to temper inflammatory cytokine production, rejuvenate cellular immune function in patients subjected to radical gastrectomy for gastric cancer, modulate inflammatory cascades, and curtail postoperative complications [17,18,39]. Thus, perioperative enteral immunonutrition significantly elevates the clinical and immunological prognosis of patients undergoing definitive gastrectomy for gastric cancer.

However, our study has several limitations. Being a single-center investigation with a circumscribed sample size, the outcomes warrant validation from larger, multicenter studies. Our follow-up duration was somewhat abbreviated, and the markers assessed were limited. An amplified sample size in future studies may encompass protracted patient monitoring, comprehensive assessment of a broader array of immunological and inflammatory markers, and meticulous scrutiny of nutritional status improvements.

## 5. Conclusions

First, this study found that using Enteral Immune Nutrition (EIN) in the perioperative period significantly reduced the levels of endotoxins and DAO on the 3rd and 7th postoperative days compared to the control group.

Second, the correlation between endotoxin levels and the NLR inflammatory marker indicates that it decreases systemic inflammatory levels by improving the intestinal mucosal barrier.

Third, our data indicated lower postoperative D-lactic acid levels in the study group compared to the control group.

Fourth, the current study found a significant moderate, positive correlation between complications, diamine oxidase on the 3rd postoperative day, and intracellular endotoxin levels.

For those undergoing radical gastrectomy as a therapeutic intervention for gastric cancer, the perioperative administration of enteral immunonutrition may bolster the intestinal mucosal barrier function, mitigate systemic inflammatory onslaughts, amplify cellular immune function, reduce postoperative complications, and accelerate patient recovery.

## Figures and Tables

**Table 1:** The comparison of general characteristics between the two groups.

Data	Study Group	Control Group	t/Chi-Square	*p*
Gender				
Male	17	27	3.097	0.078
Female	13	8		
Age (means ± SD)	61.52 ± 9.27	62.51 ± 9.63	−0.400	0.690
BMI (kg/m^2^)	23.6 ± 2.25	24.1 ± 2.73	−0.387	0.702
Tumor staging				
1	4	9	1.703	0.636
2	10	10		
3	12	11		
4	4	5		
Surgical method			0.001	0.969
Distal gastrectomy	17	20		
Total gastrectomy	13	15		
Preoperative CD4+T	693.01 ± 211.85	667.82 ± 137.23	0.559	0.578
Preoperative CD8+T	404.89 ± 114.59	405.52 ± 142.05	−0.019	0.985

Abbreviations: SD, standard deviation; BMI, body mass index.

**Table 2:** Comparison of DAO, D-lactic acid, and endotoxin between the two groups after operation.

Indicators of Intestinal Mucosal Barrier Function	Time Point	Study Group	Control Group	t	*p*
DAO	Preoperative	5.28 ± 1.53	6.19 ± 2.99	−1.572	0.122
	3rd postoperative day	5.64 ± 1.75	7.01 ± 3.07	−2.244	0.029
	7th postoperative day	4.46 ± 1.27	5.81 ± 2.77	−2.583	0.013
D-lactic acid	Preoperative	8.01 ± 2.68	9.24 ± 2.38	−1.950	0.056
	3rd postoperative day	9.00 ± 2.59	10.33 ± 2.59	−2.051	0.044
	7th postoperative day	7.02 ± 2.80	8.38 ± 2.06	−2.192	0.033
Endotoxin	Preoperative	11.77 ± 2.84	12.92 ± 2.60	−1.709	0.092
	3rd postoperative day	12.66 ± 2.91	14.43 ± 2.48	−2.645	0.010
	7th postoperative day	10.30 ± 2.61	12.23 ± 2.51	−3.027	0.004

**Table 3:** Comparison of postoperative rehabilitation between the two groups.

Groups	Time to First Flatus (Days)	Time to First Bowel Movement (Days)
Study group	2.88 ± 0.83	4.40 ± 1.15
Control group	3.57 ± 1.24	5.51 ± 1.90
t	−2.578	−2.605
*p*	0.012	0.012

**Table 4:** Comparison of postoperative complications between the two groups and correlation analysis between the complications and DAO, ET.

		Group		Chi-Square	*p*	DAO on the 3rd Postoperative Day	ET on the 3rd Postoperative Day
		Study Group	Control Group				
Complications	Without	25	21	4.252	0.039	0.335 **	0.445 **
	With	5	14				

** *p* < 0.01; Notes: DAO, diamine oxidase; ET, endotoxin.

**Table 5:** Comparison of postoperative WBC and NLR between the two groups.

Group	WBC (×10^9^/L)	NLR
Study group	6.31 ± 1.41	3.67 ± 1.84
Control group	7.73 ± 2.70	5.44 ± 4.04
t	−2.390	−2.210
*p*	0.020	0.031

Notes: WBC, white blood cell count; NLR, neutrophil-to-lymphocyte ratio.

**Table 6:** Comparison of postoperative ALB between the two groups.

Group	ALB
Study group	38.67 ± 3.38
Control group	36.46 ± 2.82
t	2.874
*p*	0.006

Notes: ALB, albumin.

**Table 7:** Comparison of postoperative CD4+T and CD8+T between the two groups.

Group	CD4+T(/μL)	CD8+T(/μL)	CD4+T/CD8+T
Study group	894.93 ± 222.68	582.23 ± 159.84	1.40 ± 0.92
Control group	719.56 ± 217.35	451.97 ± 211.44	1.78 ± 0.69
t	3.050	2.594	−1.912
*p*	0.003	0.012	0.060

## Data Availability

The datasets used and/or analyzed during the current study are available from the corresponding author on reasonable request.
