# Correction: Ma et al. Perioperative Enteral Immunonutrition Support for the Immune Function and Intestinal Mucosal Barrier in Gastric Cancer Patients Undergoing Gastrectomy: A Prospective Randomized Controlled Study. *Nutrients* 2023, *15*, 4566

**DOI:** 10.3390/nu16223800

**Published:** 2024-11-06

**Authors:** Mingwei Ma, Zicheng Zheng, Ziyang Zeng, Jie Li, Xin Ye, Weiming Kang

**Affiliations:** Department of General Surgery, Peking Union Medical College Hospital, Chinese Academy of Medical Science and Peking Union Medical College, Beijing 100730, China

In the original publication [1], there was a mistake in the following text: “Institutional Review Board Statement: Registration number was ChiCTR2300071079” as published. As this is a study on special medical food research, and not a drug clinical trial, usually there is no need to obtain a trial number; we mark and explain this in the limitation part of the article. However, in the published version [1], the registration number was marked in the Institutional Review Board Statement. The authors apologize for this misunderstanding and would like to delete the registration number in the article. The corrected version of the “Institutional Review Board Statement” appears below.

**Institutional Review Board Statement:** The present study was approved by the Institutional Review Board of the Peking Union Medical College Hospital. The ethical approval code of this study is No. KS2022530. The date of approval is 9 February 2022.

The authors state that the scientific conclusions are unaffected. This correction was approved by the Academic Editor. The original publication has also been updated.
